# Intensity matters: high-intensity interval resistance exercise induces greater immune activation than low-intensity resistance exercise 45 min post-workout

**DOI:** 10.1007/s00421-026-06337-z

**Published:** 2026-07-16

**Authors:** Fabian Fabritius, Matthias Rißmayer, Miriam Ringleb, Niklas Joisten, Wilhelm Bloch, Finn Dreisigacker, Florian Javelle

**Affiliations:** 1 NeuroPsychoImmunology Research Unit, Department for Molecular and Cellular Sports Medicine, Institute for Cardiovascular Research and Sports Medicine, German Sport University Cologne, Am Sportpark Müngersdorf 6, 50933 Cologne, Germany; 2 Department of Psychiatry and Psychotherapy, Faculty of Medicine and University Hospital of Cologne, University of Cologne, Cologne, Germany; 3 Department of Sports Medicine and Health Promotion, Friedrich Schiller University Jena, Jena, Germany; 4 Center for Interdisciplinary Prevention of Diseases Related to Professional Activities, Friedrich Schiller University Jena, Jena, Germany; 5 Research Group “Sports Medicine”, Institute for Sport and Sport Science, Technical University Dortmund, Dortmund, Germany; 6 Department for Molecular and Cellular Sports Medicine, Institute for Cardiovascular Research and Sports Medicine, German Sport University Cologne, Cologne, Germany; 7 Department of Endocrinology, Diabetology and Clinical Nutrition, University Hospital Zurich (USZ) andUniversity of Zurich (UZH), Zurich, Switzerland

**Keywords:** Resistance exercise, Exercise intensity, Neutrophil to lymphocyte ratio, Platelet to lymphocyte ratio, Systemic immune-inflammation index, Inflammation

## Abstract

**Purpose:**

Acute physical exercise triggers a transient immune response. For endurance exercise, these effects are intensity-dependent. In contrast, the role of exercise intensity in resistance exercise (RE) and its contribution to immune modulation remains less understood. This randomised crossover trial aims to compare the acute effects of High-Intensity Interval Resistance Exercise (HIIRE) versus Low-Intensity Resistance Exercise (LIRE) on routine clinical cellular inflammatory markers—neutrophil-to-lymphocyte ratio (NLR), platelet-to-lymphocyte ratio (PLR), and systemic immune-inflammation index (SII)—in 24 healthy, RE-trained young men.

**Methods:**

Measurements were taken at baseline (T_0_), immediately post-exercise (T_1_), and 45 min post-exercise (T_2_). It was hypothesised that HIIRE would elicit a significantly greater increase in NLR, PLR, and SII from baseline (T_0_) to post-exercise (T_1_ and T_2_) compared to LIRE.

**Results:**

Baseline-adjusted ANCOVAs revealed significant interactions for cellular inflammatory markers (*η*^2^_p_ = .072; NLR: *p* < .001, *η*^2^_p_ = .217; PLR: *p* < .001, *η*^2^_p_ = .147; SII: *p* < .001, *η*^2^_p_ = .204). Post hoc analyses locate this difference for NLR, PLR, and SII 45 min post-exercise, with greater increases of HIIRE.

**Conclusion:**

The results suggest a complex immune response involving pro-inflammatory signalling and immune cell redistribution induced by acute RE, with intensity being a main driver. This underscores the pivotal role of intensity in modulating acute immune responses to RE, with potential implications for exercise prescription targeting inflammatory processes.

**Supplementary Information:**

The online version contains supplementary material available at https://doi.org/10.1007/s00421-026-06337-z.

## Introduction

Regular physical exercise is well established to enhance the body’s anti-inflammatory capacity and metabolic health (Gleeson et al. 2011; Ortega 2016). These long-term adaptations are driven by repeated single exercise bouts, each eliciting a transient pro-inflammatory response and immune activation (Ringleb et al. 2024, 2026) followed by a remission phase (Gleeson et al. 2011). The acute response is characterised by temporary alterations in the distribution of white blood cells (WBCs) and increased concentrations of circulating cytokines (collectively known as Exerkine), primarily myokines produced by contracting muscles, such as interleukin-6 (IL-6) (Shek et al. 1995; Pedersen et al. 1998).

It was first demonstrated that sympathetic activation during physical exercise, via catecholamine release, induces demargination of WBCs (particularly neutrophils and lymphocytes) from the vascular endothelium into the circulation (Nieman 1997). This immediate response is followed, with some delay, by increased cortisol levels, which further modulate immune cell trafficking and exert immunosuppressive properties, contributing to the dose-dependent "open window effect" (Kakanis et al. 2010; Jones and Davison 2019). More recently, it has been recognised that other tissues can produce signalling molecules (Exerkines) in response to physical exercise, which play an important role in shaping the acute immune response (Chow et al. 2022). This is especially true for skeletal muscle, where contractions stimulate the release of myokines (Muñoz-Cánoves et al. 2013; Chow et al. 2022), which not only drive a transient pro-inflammatory phase but also help meet metabolic demand and initiate anti-inflammatory cascades (Pedersen and Fischer 2007; Muñoz-Cánoves et al. 2013). For example, the skeletal muscle-derived IL-6 is primarily produced in response to changing energy demands (Muñoz-Cánoves et al. 2013; Lin et al. 2023). The resulting increase in IL-6 promotes glycogen breakdown to support ATP synthesis during exercise (Kistner et al. 2022). Beyond metabolism, muscle-derived IL-6 exerts anti-inflammatory effects by modulating neutrophil mobilisation and recruitment (Yamada et al. 2002) and inducing a post-exercise anti-inflammatory response via upregulation of IL-10, IL-1 receptor antagonist, and regulatory T cells (Petersen and Pedersen 2005; Ringleb et al. 2024, 2026).

When examining the acute effects of exercise on routinely measured immune cells and associated factors, several characteristic responses can be observed. Neutrophils, indexing for the innate immune system, increase both during and after exercise to support tissue repair (Brown et al. 2015; Kawanishi et al. 2016). Lymphocytes, representing the adaptive immune system, transiently increase during exercise but rapidly decrease thereafter, a response associated with elevated IL-6 and cortisol levels, as well as the activation of one of their smallest subsets, regulatory T cells (Pedersen and Steensberg 2002; Steensberg et al. 2003). Platelets, known for their role in haemostasis, have also emerged as active regulators of immune processes (Ribeiro et al. 2019; Maouia et al. 2020; Nicolai et al. 2024). During acute exercise, platelet count increases via multiple mechanisms (e.g., splenic contraction, catecholamine-mediated activation, endothelial activation, haemoconcentration) (Mosso-Pani et al. 2025) and interacts with WBCs, particularly neutrophils, to contribute to the coordinated immune activation and tissue remodelling that characterise the early post-exercise period (Mosso-Pani et al. 2025).

To capture all these responses, routine composite indices have gained clinical relevance due to their integrative capacity, low cost, and ease of calculation (Walzik et al. 2021). The most commonly used are the neutrophil-to-lymphocyte ratio (NLR), platelet-to-lymphocyte ratio (PLR), and systemic immune-inflammation index (SII) (Walzik et al. 2021). The exercise-induced increase in NLR reflects the balance between innate immune activation and adaptive immune suppression (Song et al. 2021), whereas an elevated PLR indicates heightened thrombotic activity during vascular inflammation accompanied by adaptive immune suppression (Wang et al. 2023). While NLR serves as a marker of stress-related immune shifts, PLR captures thrombo-inflammatory processes beyond NLR’s scope. Finally, the SII integrates these immune shifts by combining NLR and platelet counts, providing a comprehensive measure of systemic immune activation.

A large body of scientific data has demonstrated that acute endurance exercise (EE) elicits a transient pro-inflammatory response in a dose-dependent (intensity × duration) manner (Ringleb et al. 2024, 2026). Although a similar pro-inflammatory response has been demonstrated in resistance exercise (RE), establishing its dose dependency, especially regarding intensity, remains challenging due to the methodological heterogeneity and variation across existing studies (Ringleb et al. 2024). A review of Walzik and colleagues from 2021 (Walzik et al. 2021) showed that there is robust evidence that acute EE leads to a significant increase in routine immune composite indices in an intensity-dependent manner, with NLR being the one most evidences (Nieman et al. 1995; Davison and Diment 2010; Murase et al. 2016; Wei et al. 2017; Korkmaz et al. 2018; Wahl et al. 2020) followed by PLR (Korkmaz et al. 2018; Wahl et al. 2020) and SII (Wahl et al. 2020; Schlagheck et al. 2020). However, because previous research on RE has focused almost exclusively on single high-volume eccentric bouts (Kerksick et al. 2010; Joisten et al. 2019) which showed significant increases in NLR and SII, and no trial investigated the modulatory role of intensity in acute RE, this randomised crossover trial investigated how high- versus low-intensity RE affects routine composite immune indices in healthy young men.

RE intensity can be manipulated by many factors, such as increasing external load (load-based intensity) or by prolonging time under tension or shortening rest intervals (effort-based intensity). The cluster training method has been shown to be particularly effective in increasing time at high effort and high load (Tufano et al. 2017), driving up metabolic demand and immune response. By inserting brief intra-set recovery periods, this configuration allows partial phosphocreatine resynthesis and metabolite clearance, enabling greater total workload per set and greater velocity maintenance than traditional continuous-set RE (Lawton et al. 2004; Haff et al. 2008). In the High-Intensity Interval Resistance Exercise (HIIRE) cluster paradigm, a single all-out set to momentary muscular failure (MMF; i.e., point during a set when another full repetition with proper technique despite maximal effort cannot be completed) is followed by two cluster sets, each to MMF and separated by brief passive intra-set recovery intervals of 20 s (Paoli et al. 2012). This approach prolongs the time spent at or near maximal intensity within a set compared to traditional RE modalities that employ only a single effort per set. A single bout of HIIRE elicits a potent acute metabolic and immunological response, which, if repeated over time, may potentially induce sustained metabolic and immunological adaptations that promote an anti-inflammatory environment and improved metabolic health (Calle and Fernandez 2010).

Therefore, this randomised crossover trial investigated the acute effects of HIIRE on immune activation in healthy, RE-trained young men. Specifically, the study examined how exercise intensity modulates routine immune composite indices, specifically, the NLR, PLR, and SII. The effects of a single bout of HIIRE were compared to a single bout of Low-Intensity Resistance Exercise (LIRE) at three time points: baseline (T_0_), immediately post-exercise (T_1_), and 45 min post-exercise (T_2_), in healthy, RE-trained young men. It was hypothesised that HIIRE would elicit a significantly greater increase in NLR, PLR, and SII from baseline (T_0_) to post-exercise (T_1_ and T_2_) compared to LIRE.

## Materials and methods

All methods contributing to this work comply with the ethical standards of the responsible national and institutional committees for human experimentation and with the Declaration of Helsinki of 1975, as amended in 2008. After ethical approval, the protocol of this trial with directional hypotheses together with raw data was uploaded to Open Science Framework (URL: https://doi.org/10.17605/OSF.IO/E75N9). This paper presents the detailed procedures and secondary outcomes of the study. The primary outcomes (kynurenine metabolites) are reported in Javelle et al. (*in preparation*).

### Experimental design

**Fig. 1:**
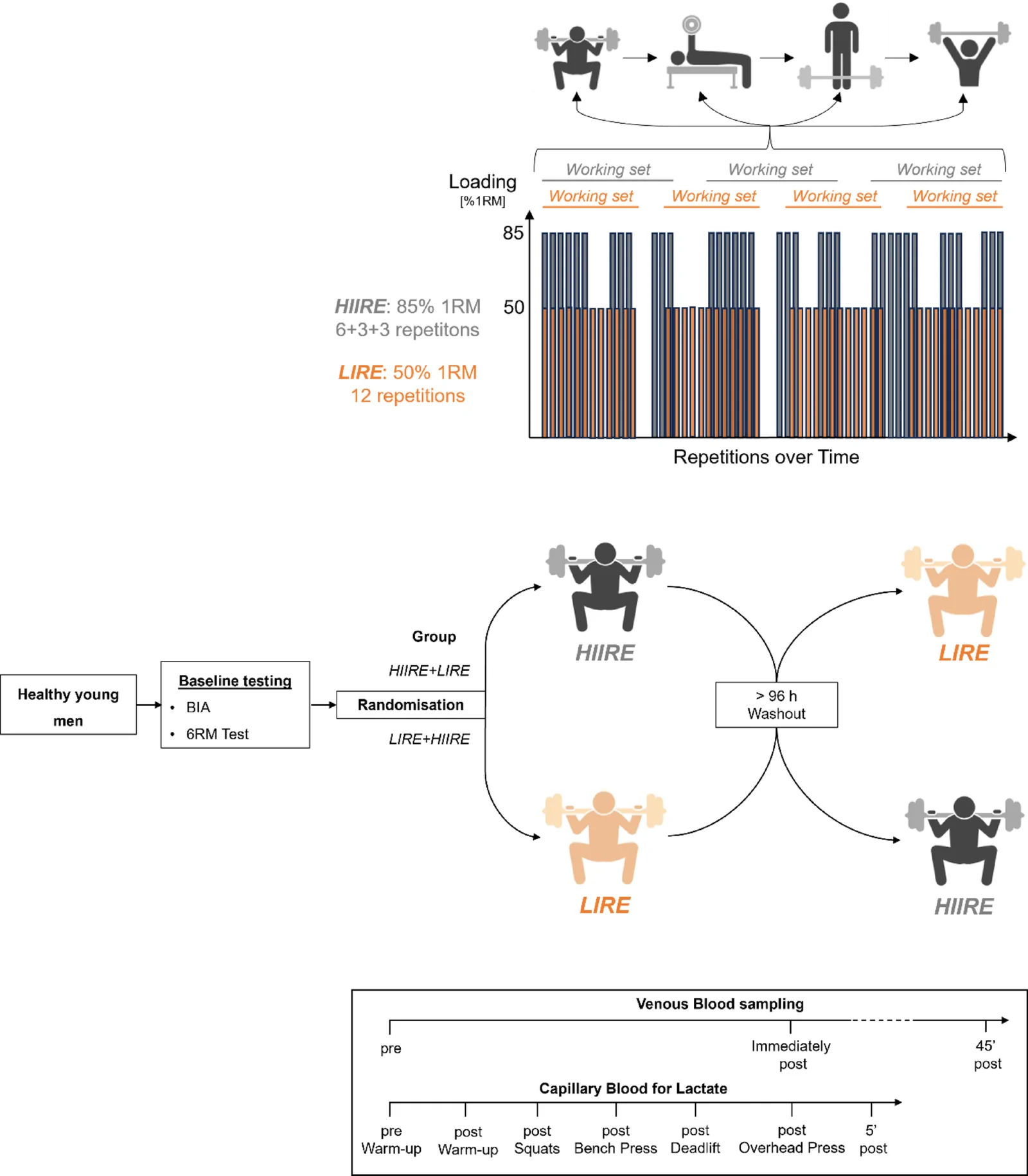
Experimental design. *BIA*: Bioelectrical Impedance Analysis, *h*: hours, *HIIRE:* High-Intensity Interval Resistance Exercise, *LIRE*: Low-Intensity Resistance Exercise, *RM:* repetition maximum; ': minutes. Image created with BioRender.com

Participants were recruited through an online screening questionnaire hosted on the Qualtrics platform. Recruitment strategies included word of mouth, announcements on the University’s website, and social media posts. Before the trial began, participants received detailed verbal and written explanations of the trial protocol. Informed consent was obtained online before completing the screening questionnaire and was confirmed in writing during the first in-person meeting. During this initial meeting, participants were introduced to the study tools and procedures, inclusion and exclusion criteria were verified, and preliminary measures were assessed (e.g., six-repetition maximum (6RM) strength test). Participants were then randomly assigned to two different intervention sequence groups: Group A (HIIRE + LIRE) and Group B (LIRE + HIIRE). They attended the following interventions: (i) a bout of HIIRE and (ii) a bout of LIRE, separated by a washout period (> 96 h) to avoid carry-over effects. Venous blood was sampled at baseline T_0_), immediately (T_1_), and 45 min post-exercise (T_2_) (Fig. 1). Participants were blinded to the trial’s hypotheses.

### Participants

The inclusion criteria were being male, aged 18 to 30, and RE-trained. The degree of experience in RE was indicated by the participants during the screening process via a visual analogue scale (from 1 (no experience) to 10 (highly experienced); inclusion: ≥ 5 (recreationally experienced)) and then verified at the familiarisation session during the 6RM test based on the exercise execution quality. For homogeneity purposes, only men were examined. The main exclusion criteria were acute infections or injuries, as well as chronic diseases such as psychiatric, metabolic, renal, hormonal, intestinal, neuroendocrine, or chronic infectious and inflammatory diseases, motor and myopathic disorders, and cardiovascular problems. Exclusion thresholds for nicotine and alcohol use were set to usage > 20 cigarettes per day in the last three months and not having a high risk for potential alcohol dependency (Alcohol Use Disorders Identification Test (AUDIT) sum score > 19 (Babor et al. 2001)). Current smokers below this threshold were not excluded to preserve external validity in an otherwise healthy resistance-trained cohort. However, participants were required to abstain from smoking throughout the testing days until completion of the final measurement. Participants who used medication were required to maintain consistent intake throughout the trial and have a stable dosage at least 7 days before the start of the experiment. Participants who consumed performance-enhancing drugs were excluded. Participants were asked not to consume tryptophan-containing supplements (e.g., tryptophan, 5-Hydroxytryptophan, or essential amino acid supplements) at least 7 days before the familiarisation session and throughout the trial.

### Sample size

Sample size was calculated based on a moderate *Time* × *Condition* interaction of the kynurenic acid / kynurenine ratio (index of kynurenine aminotransferases activity) on a baseline-adjusted ANCOVA (for details, Javelle et al., *in preparation*). Based on the parameters used, the required sample was 47 participants. Given the crossover design, in which each participant completed both trial arms, the effective sample size was n = 23.5; therefore, N = 24 participants were targeted to balance intervention sequences. A posteriori power analyses were conducted for changes in routine immune composite indices (*simr* package).

### Randomisation

Randomisation was carried out immediately after the familiarisation session using the package Minirand on the software R. A randomisation procedure according to Pocock and Simon’s minimisation method (Scott et al. 2002) was used. Minimisation factors comprised participants’ fat-free mass index (FFMI) (< 20: low to normal muscle mass; ≥ 20: muscular above average; ≥ 22 very muscular)(Schutz et al. 2002; Chwałczyńska et al. 2022) and total 6RM strength level (Reeves et al. 2004). The latter was determined by a strength test to obtain the individual and exercise-specific 6RM, and was calculated using a total score for all four exercises. The 6RM strength level (Section "6-repetition maximum test") for each of the four exercises was divided into three ascending levels based on the percentage of individual strength value relative to body mass (Santos Junior et al. 2021), with one to three points awarded from bottom to top for each exercise. This resulted in a total score ranging from 4 to 12 points, divided into three categories: 4–6, 7–9, and 10–12 (Supplementary Material 1: Tables S1, S2 and S3).

### Physical strength and body composition assessment

#### 6-repetition maximum test

To determine individualised training loads for the HIIRE and LIRE *Conditions*, participants performed a standardised 6RM assessment. Because the HIIRE protocol requires ~ 85% 1RM, which is equivalent to ~ 100% 6RM, the 6RM test was chosen as a safe and reliable method for evaluating maximal strength (Reeves et al. 2004). For each exercise (barbell back squat, guided barbell flat bench press, barbell Romanian deadlift, and barbell standing overhead press), participants first estimated a realistic target to plan and then tested it during the first in-person meeting. Exercise technique and range of motion were standardised across participants (Supplementary Material 1), and all movements were required to be performed under full control and without momentum-assisted or ballistic patterns (Supplementary Material 1: Table S2).

#### Bioelectrical impedance analysis

Bioelectrical Impedance Analysis (BIA) has proven to be a reliable, fast, and accurate scientific tool for assessing body composition (Silva et al. 2023). The measurement was conducted after an overnight fast. Participants were asked to drink approximately 500 mL of water within the first hour after waking up and to refrain from drinking water for at least 45 min before the measurement. Additionally, participants were required to empty their bladders immediately before the measurement. Prior to initiating the measurement process, participants were required to assume a supine position with their legs slightly apart and their arms extended for a period of 10 min. After attaching two electrodes (BIATRODES™) to the right side of the body, one on each hand and foot, and connecting them to the BIA device (phase-sensitive system, 50 kHz BIA 101 RJL, Akern Bioresearch, Florence, Italy), the measurement was started to get BIA-based body resistance (RZ) values, together with reactance (XC) values. Then those values were imported into BODYGRAM (Akern) software together with participants’ biological sex, body mass, and height to extract their fat mass, fat-free mass, and compute the FFMI.

### Interventions

Participants came after an overnight fast (8-12 h, no supplementation) and were allowed to consume only water during each exercise *Condition*. Prior to each exercise bout, participants were required to refrain from RE for a minimum of 72 h and EE for a minimum of 48 h. Additionally, participants were asked to refrain from consuming caffeine, medication, and alcohol for 24 h prior to each intervention. On the day of each intervention, participants were instructed to minimise physical activity and stress to the greatest extent possible. Interventions were scheduled at the same time of day (± 60 min, between 7 a.m. and 11 a.m.) to ensure comparability. If participants exhibited symptoms suggestive of an acute infection on the day of intervention, appointments were rescheduled. Participants executed a standardised warm-up protocol, starting with 5 min on a bicycle ergometer at 30 watts (cadence 60–90 rpm), followed by 5 min of dynamic mobilisation. Immediately after finishing the warm-up, participants began the RE *Condition*. Exercises were performed in the following order: 1) barbell back squat, 2) guided barbell flat bench press, 3) barbell Romanian deadlifts, and 4) barbell standing overhead presses. Inter-set rest periods were standardised to two minutes between working sets for both interventions. Rest periods were strictly controlled and adhered to. HIIRE and LIRE were designed to be duration- and relative volume-matched. Duration was defined as the time from the first warm-up set of the initial exercise (the squat) to the final repetition of the session. Relative volume was calculated as the total number of repetitions multiplied by the percentage of 1RM used (Cavarretta et al. 2018; for example, in LIRE: 4 sets × 12 reps × 4 exercises × 0.50 1RM = 96).

#### High-intensity interval resistance exercise

First, three exercise-specific warm-up sets were performed, with participants using the empty barbell (20 kg) at 40% and 60% of their 1RM, for 12, 10, and 8 repetitions, respectively. Subsequently, participants performed three working sets per exercise. For each exercise, one working set consisted of 6 repetitions with ~ 85% 1RM until MMF, 20 s passive intra-set rest, 2–3 repetitions until MMF, 20 s passive intra-set rest, 2–3 repetitions until MMF (Fig. 1). To achieve maximal intensity and ensure the targeted intervention volume was standardised, load adjustments were made during the intervention. If a participant completed more than 14 reps in the working set, the load was increased by 10% for the next set. If they completed fewer than 10 reps, the load was reduced by 10%.

#### Low-intensity resistance exercise

Participants performed three exercise-specific warm-up sets with the empty barbell (20 kg), at 30% and 40% of their 1RM, with 12, 10, and 8 repetitions, respectively. For each exercise, one of the four working sets consisted of 12 repetitions performed under continuous muscular tension at ~ 50% 1RM (Fig. 1). In the rare cases where participants were unable to complete 12 repetitions, the load was reduced by 10% for the next working set to reach the target repetitions.

### Blood collection and analyses

#### Cellular immune-inflammation markers

Venous blood was sampled on the antecubital vein in one 10 ml EDTA vacutainer at baseline (T_0_), immediately (T_1_), and 45 min post-exercise (T_2_). Complete blood count (KX-21N, Sysmex Europe, Norderstedt, Germany) was performed immediately after sample collection as routine control measurements. Subsequently, NLR, PLR, and SII were computed based on complete blood counts for lymphocytes, neutrophils and platelets (Supplementary Material 1: Table S4).

#### Capillary lactate

Capillary blood was drawn from the earlobe with a sterile lancet at seven time points (pre-warm-up, post-warm-up, post-squat, post-bench press, post-deadlift, post-overhead press, and 5 min post-exercise—Fig. 1). The levels of capillary lactate (cLac) were measured with the Biosen S-Line (EKF Diagnostics) in order to provide descriptive characteristics for each exercise *Condition*.

### Heart rate measurement

To measure the heart rate (HR), a fitness tracker watch (Polar Vantage M2) connected to a wireless chest sensor (Polar H9) was used for each participant in both *Conditions*. HR was measured throughout the entire intervention, starting with the RE warm-up and continuing for 5 min post-exercise (Section "Manipulation check"; Supplementary Material 1: Table S6).

### Statistical analysis

Statistical analyses and representations were performed using JASP (Version 0.19.1.0) and R (version 4.5.0). All cell counts were corrected using the Dill and Costill formula (Dill and Costill 1974) to account for changes in plasma volume induced by exercise. Data were then winsorised at ± 3 standard deviations (SDs; 1.37% of all data points). One missing LIRE T_2_ blood draw was imputed. To do so, the percentage change of the corresponding HIIRE values from T_1_ to T_2_ was calculated for each variable of interest and transformed into a z-score relative to the HIIRE mean. Thereafter, the imputed LIRE values were obtained by applying the computed z-score to the LIRE mean percentage change from T_1_ to T_2_ for each variable. Normal distribution was assessed using the Shapiro–Wilk test. Furthermore, all variables were visually checked for linearity (using quantile–quantile plots and histograms of standardised residuals), as well as for skewness and kurtosis. Shapiro–Wilk tests for neutrophils, NLR, PLR, and SII revealed significant deviations from normality and positive kurtosis. Thus, the logarithm to base 10 (log_10_) transformation was applied to these variables. After the transformation, the data were normally distributed. For data visualisation and presentation in the manuscript, a back-transformation was applied. Participants’ characteristics were tested for baseline Group differences using Independent Samples T-Tests and Chi-Squared (*Χ*^*2*^) tests. Manipulation checks on HR_mean_ were tested using Paired Samples T-Tests. Baseline-adjusted mixed-effect ANCOVAs (*package lme4*) were conducted to evaluate the effects of the exercise *Condition* on cLac, cell counts (i.e., neutrophils, lymphocytes, platelets), and routine immune composite indices (i.e., NLR, PLR, SII). Sphericity was assessed using Mauchly’s test, and violations were corrected with the Greenhouse–Geisser adjustment; homogeneity of variance was verified with Levene’s test. Significant effects were further specified using post hoc comparisons with Bonferroni correction, adjusting for multiple testing using R (packages *afex* and *emmeans*). The significance level was set to .05. Significant effects between *Conditions *or* Timepoints* were reported using covariate-adjusted within-subject Cohen’s *d* (using the combined random-intercept and residual variance from the mixed-effects model to avoid effect sizes inflation).

## Results

### Participant description

**Fig. 2:**
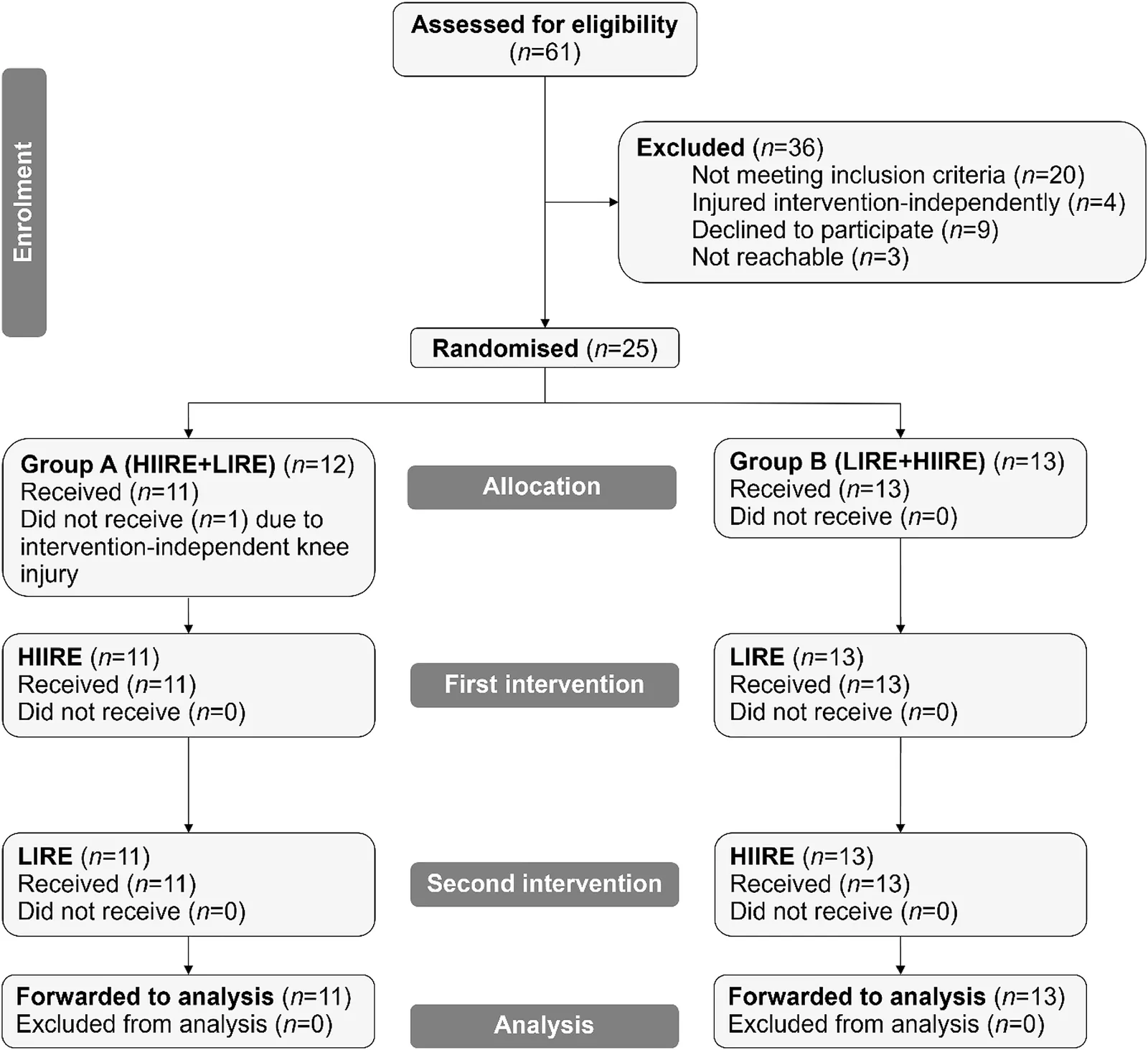
CONSORT participant flowchart.* HIIRE* High-Intensity Interval Resistance Exercise,* LIRE* Low-Intensity Resistance Exercise

As shown in the Consolidated Standards of Reporting Trials (CONSORT) flowchart (Fig. 2), 25 participants were randomised. One participant dropped out due to an intervention-independent injury between the familiarisation session and the first intervention. For the whole trial, no adverse events were observed. Total and Group-specific demographic and anthropometric characteristics of participants are displayed in Table 1. No significant *Group* (i.e., sequence order) differences were detected, nor for minimisation factors (i.e., FFMI and total 6RM score; Table 1), nor for age, body mass, height, fat mass, fat-free mass, as well as the AUDIT score indexing the success of the randomisation.

**Table 1:** Total and Group-specific demographic and anthropometric characteristics of participants

	Total	HIIRE + LIRE	LIRE + HIIRE	Independent Samples T-Test
	*n* = 24	*n* = 11	*n* = 13	*p*
Age	23.50 ± 2.90	23.00 ± 2.10	23.80 ± 3.40	.491
Body mass [kg]	83.85 ± 10.09	86.62 ± 9.87	81.52 ± 10.05	.225
Height [m]	1.82 ± 0.07	1.82 ± 0.08	1.81 ± 0.07	.694
Fat mass [kg]	15.22 ± 6.00	16.77 ± 4.59	13.91 ± 6.87	.252
Fat mass [%]	17.82 ± 5.78	19.19 ± 3.75	16.67 ± 7.01	.297
Fat-free mass [kg]	68.19 ± 7.13	69.06 ± 7.15	67.45 ± 7.32	.595
FFMI [kg · m^−2^]	20.65 ± 1.76	20.75 ± 1.59	20.57 ± 1.96	.801
Total 6RM Score (min. 4 – max. 12)	8.8 ± 1.6	8.6 ± 1.4	8.9 ± 1.7	.664
AUDIT score	5.60 ± 3.60	5.90 ± 3.50	5.30 ± 3.80	.693
Current smoker	3 (12.50%)	1 (9.10%)	2 (15.40%)	.461*

Means ± SDs are presented

*HIIRE* High-Intensity Interval Resistance Exercise, *LIRE* Low-Intensity Resistance Exercise, *AUDIT* Alcohol Use Disorders Identification Test, *FFMI* Fat-Free Mass Index, *RM* Repetition Maximum

*Group difference was tested with a Chi-Squared Test

The sequence allocation did not induce differences for duration (*p* = .129; Section "Interventions"; Supplementary Material 1: Table S5) or relative volume (*p* = .098; Section "Interventions"; Table S5). No significant *Condition* differences (i.e., HIIRE vs LIRE) were detected for relative volume (*p* = 0.691). Although, session duration was significantly longer in HIIRE (*p* < .001), the difference amounted to only 3 min 24 s across sessions lasting approximately 80 min, including warm-up (Supplementary Material 1: Table S5) (see Fig. 2).

### Manipulation check

When data from the two exercise bouts were combined, sequence allocation did not show an effect on heart rate (HR_mean_). When comparing *Conditions*, HR_mean_ (*p* < .001; *d* = 0.891) was significantly higher during HIIRE (HR_mean_: 126.8 bpm) than during LIRE (HR_mean_: 110.2 bpm).

**Fig. 3:**
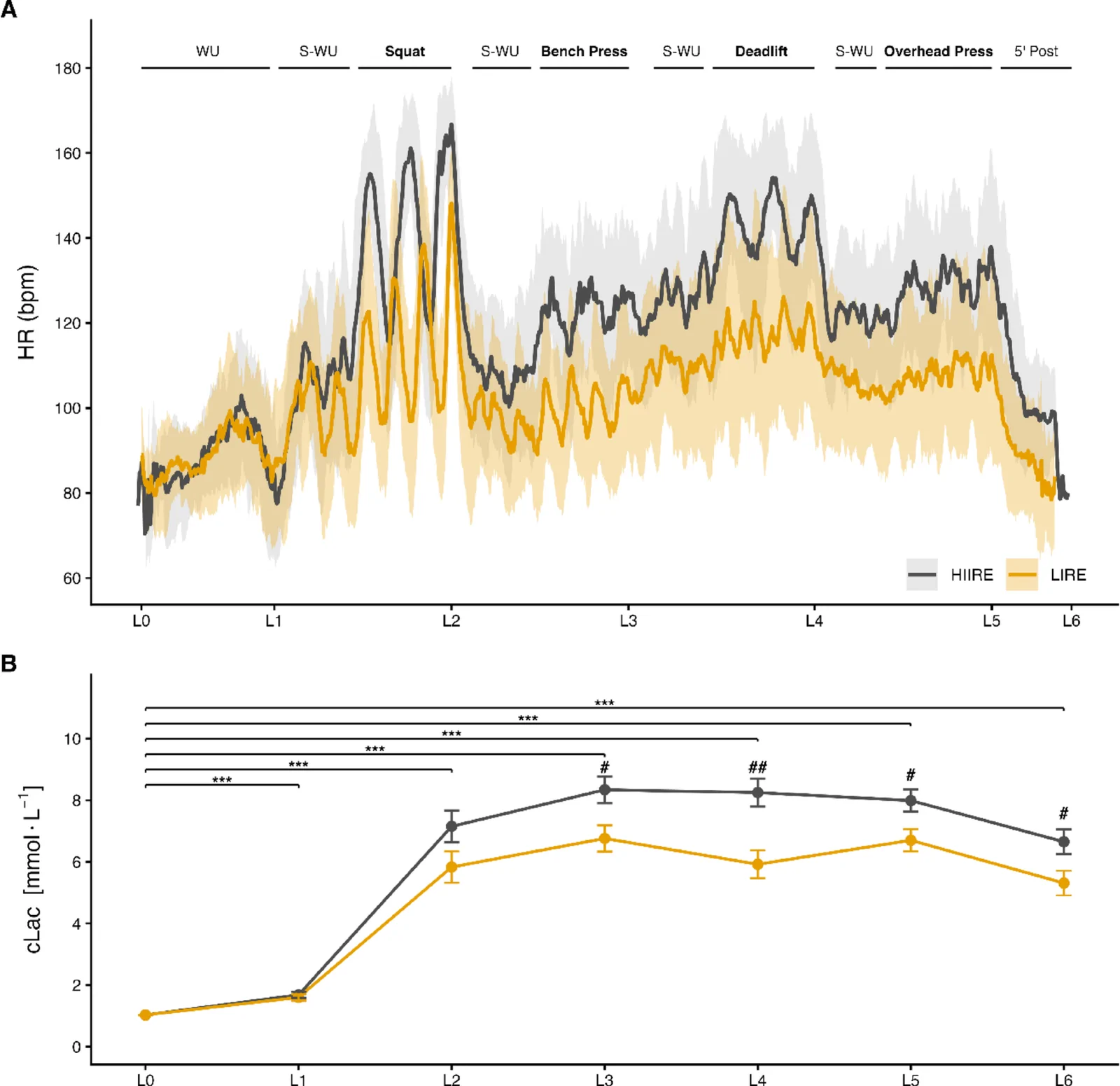
**A** Heart rate response to both exercise *Conditions*. The graphs are intended only as visual characteristics of the exercise bouts. Although the sessions were designed to be time-matched within individuals, some participants performed the exercises at markedly different rhythms (especially in the HIIRE, where a different number of repetitions could be performed before reaching muscle failure), resulting in excessive variance when plotted together. These participants were therefore excluded from the graphic. The lighter shaded areas represent the standard deviation for each Condition. Black segments indicate the sequence of the protocol. *HIIRE:* High-Intensity Interval Resistance Exercise (in grey), *LIRE*: Low-Intensity Resistance Exercise (in orange), *S-WU:* Specific Warm-Up, *WU*: Warm-Up. **B** Baseline-adjusted means for capillary lactate by *Time* and *Conditions*. The line chart shows baseline-adjusted mean capillary lactate concentrations [mmol·L⁻^1^] for HIIRE (grey) and LIRE (orange) from L0 to L6. Error bars indicate SEM. Asterisks denote significant Time effects (comparisons vs. L0): *p* < .050 (*), *p* < .010 (**), *p* < .001 (***). Hash marks indicate significant *Time* × *Condition* interactions: *p* < .050 (#), *p* < .010 (##), *p* < .001 (###). Time points: *L0* pre warm-up, *L1* post warm-up, *L2* post Squat, *L3* post Bench Press, *L4* post Deadlift, *L5* post Overhead Press, *L6* 5 min post-exercise

For cLac, as expected, sequence allocation did not show an effect (Supplementary Material 1: Table S6). Baseline-adjusted repeated-measures ANCOVAs revealed significant *Time* × *Condition* interactions with a moderate effect size (*p* = .004; *η*^*2*^_*p*_ = .093). As displayed in Fig. 3, both exercise *Conditions* have shown significant lactate increase from L2 (post-Squat) and sustained until the last measure L6 (5-min post-exercise). Post hoc analyses also displayed significant moderate to large *Condition* differences in favour of HIIRE at L3 (*d* = 0.538; *p* = .013), L4 (*d* = 0.749; *p* = .001), L5 (*d* = 0.518; *p* = .014), and L6 (*d* = 0.483; *p* = .023).

### High-intensity interval resistance exercise induces stronger increases in cellular inflammation markers

As expected, the sequence allocation did not influence cellular inflammatory response to exercise (Table 2).

**Table 2:** Back-transformed baseline-adjusted means of cellular inflammatory markers by *Time* and *Condition*

		HIIRE		LIRE		*Time* × *Condition*	
	*n* = *48*	*n* = 24		*n* = 24		*n* = *24*	
	T_0_	T_1_	T_2_	T_1_	T_2_	*p*	*η* ^*2*^_*p*_
NLR	1.388	1.812 ± 0.118	3.645 ± 0.196	1.767 ± 0.117	2.334 ± 0.140	< .001***	**.226**
PLR	103.713	108.648 ± 3.913	157.489 ± 5.656	116.490 ± 4.193	137.038 ± 4.927	= .002**	**.107**
SII	294.121	425.580 ± 26.913	793.328 ± 50.115	406.380 ± 25.702	511.861 ± 32.357	< .001***	**.174**

^Table displays back-transformed baseline-adjusted Means ± Standard Errors of the Means (SEMs) of cellular inflammatory markers NLR, PLR and SII^

^*HIIRE* High-Intensity Interval Resistance Exercise, *LIRE* Low-Intensity Resistance Exercise, *NLR* Neutrophil-to-Lymphocyte Ratio, *PLR* Platelet-to-Lymphocyte Ratio, *SII* Systemic Immune Inflammation Index, *T0* baseline, *T1* immediately post-exercise, *T2* 45-min post-exercise^

^Bolded and asterisked areas mark significant differences* at p* < .050 (*), *p* < .010 (**), and *p* < .001 (***)^

**Fig. 4:**
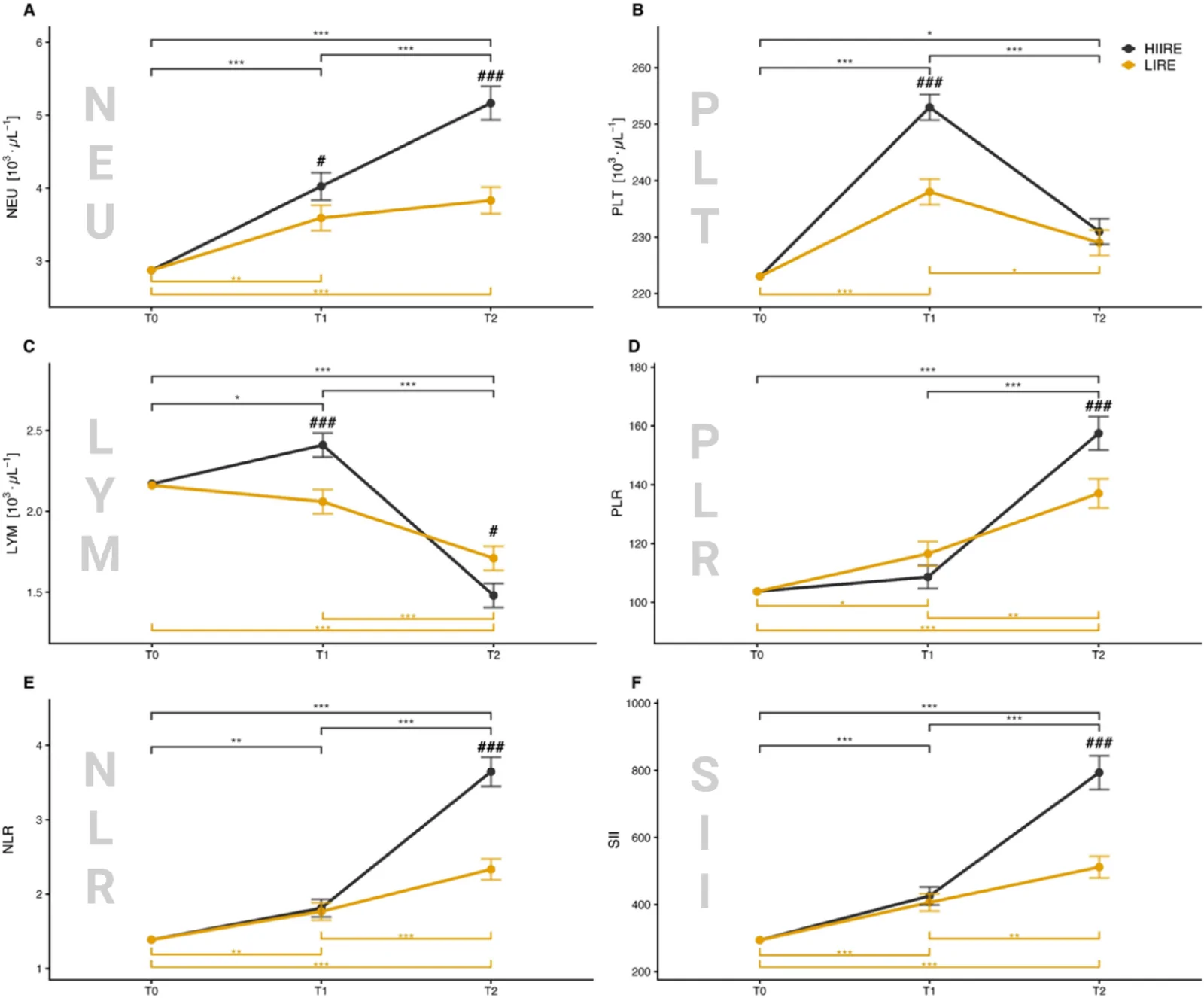
Back-transformed baseline-adjusted means for cell counts and inflammatory markers by Time and Conditions. Line charts display baseline-adjusted means of Neutrophils (**A**), Platelets (**B**), Lymphocytes (**C**) and composite markers Platelet-to-Lymphocyte Ratio [PLR] (**D**), Neutrophil-to-Lymphocyte Ratio [NLR] (E) and Systemic Immune Inflammation Index [SII] (F) for HIIRE (grey) and LIRE (orange) at T_0_, T_1_ to T_2_. Error bars represent Standard Errors of the Mean (SEMs). Asterisk signs indicate significant main effects for Time at *p* < .05 (*), *p* < .01 (**) and *p* < .001 (***) and dash signs indicate significant Time × Condition interaction at *p* < .05 (#), *p* < .01 (##) and *p* < .001 (###). *T*_*0*_: baseline, *T*_*1*_ : immediately post-exercise, *T*_*2*_: 45-min post-exercise

To ensure transparency, the individual components of the immune indices (i.e., lymphocytes, platelets, and neutrophils) were explored separately, and the results are presented in Fig. 4. Baseline-adjusted repeated measures ANCOVAs revealed significant *Time* × *Condition* interactions with moderate to large effects for platelets (*η*^*2*^_*p*_ = .121; *p* < .001), large effects for lymphocytes (*η*^*2*^_*p*_ = .155; *p* < .001), and large effects for neutrophils (*η*^*2*^_*p*_ = .145; *p* = .001). Neutrophil counts increased from pre- to immediately post-exercise in both *Conditions* but was more pronounced following HIIRE than following the LIRE, as indicated by post hoc interactions immediately post- and 45 min post-exercise (T_1_: *d* = 0.500, *p* = .023; T_2_:* d* = 1.331, *p* < .001; Fig. 4A). Platelet levels increased from pre- to post-exercise in both *Conditions*, with a significantly greater rise in the HIIRE than in the LIRE *Condition* (*d* = 1.356, *p* < .001; Fig. 4B) and came back to baseline 45 min post-exercise. For lymphocytes, post hoc detected the interactions immediately post- (HIIRE > LIRE; *d* = 0.960, *p* < .001) and 45 min post-exercise (*d* = -0.651, *p* = .011). In both *Conditions*, a lymphopenia was observed 45 min post-exercise, with more pronounced effects following HIIRE (T_1_ vs T_2_: *d* = 2.571, *p* < .001; T_0_ vs T_2_: *d* = 1.892, *p* < .001; Fig. 4C) than following LIRE (T_1_ vs T_2_: *d* = 0.960, *p* < .001; T_0_ vs T_2_: *d* = 1.226, *p* < .001)..

Then, when assessing composite indices, significant *Time* × *Condition* interactions with large effects for NLR (*η*^*2*^_*p*_ = .226; *p* < .001), moderate to large effects PLR (*η*^*2*^_*p*_ = .107; *p* = .002) and large effects SII (*η*^*2*^_*p*_ = .174; *p* < .001) were detected. As shown in Fig. 4 and Table 2, the composite indices increased from pre- to immediately post-exercise and further increased 45 min post-exercise. Post hoc analyses (Fig. 4) indicated that in the HIIRE group, NLR (*d* = 0.786; *p* = .002) and SII (*d* = 1.224; *p* < .001) increased from from pre- to immediately post- exercise; NLR (*d* = 2.425; *p* < .001), PLR (*d* = 2.117; *p* < .001) and SII (*d* = 2.015; *p* < 0.001) increased from immediately-post- to 45 min post-exercise; and NLR (*d* = 3.211; *p* < .001), PLR (*d* = 2.377; *p* < .001) and SII (*d* = 3.240; *p* < .001) increased from baseline to 45 min post-exercise. A similar pattern was observed in the LIRE group, where post hoc analyses indicated that in the LIRE group, NLR (*d* = 0.707; *p* = .006) and SII (*d* = 1.063; *p* < .001) increased from from pre- to immediately post-exercise; NLR (*d* = 0.909; *p* < .001), PLR (*d* = 0.849; *p* = .003) and SII (*d* = 0.791; *p* < .001) increased from immediately-post- to 45 min post-exercise, and NLR (*d* = 1.616; *p* < .001), PLR (*d* = 1.480; *p* < .001) and SII (*d* = 1.854; *p* < .001) increased from baseline to 45 min post-exercise.

Additionally, *Time* × *Condition* interactions were observed 45 min post-exercise in favour of the HIIRE *Condition* for NLR (*d* = 1.595; *p* < .001; Fig. 4D), PLR (*d* = 0.878; *p* = .006; Fig. 4E), and SII (*d* = 1.391; *p* < .001; Fig. 4F).

A-posteriori power analyses for each main *Time* × *Condition* interactions, along with their 95% confidence intervals (CI), are presented in Supplementary Material 1: Table S7. Overall, the power is excellent, with the lowest bounds in all 95% CIs being 88.4%.

## Discussion

The primary aim of this randomised crossover trial was to investigate the acute effects of HIIRE versus LIRE on cellular immune activation from baseline (T_0_) to immediately (T_1_) and 45 min post-exercise (T_2_) in 24 healthy young men experienced in RE. It was hypothesised that HIIRE would induce a significantly greater increase in NLR, PLR, and SII from baseline (T_0_) to post-exercise (T_1_ and T_2_) compared to LIRE. In the present study, HIIRE produced more pronounced metabolic and cardiovascular responses (cLac and HR_mean_) during exercise, leading to a greater acute increase in platelets immediately after exercise (T_1_), but without *Condition* differences in the composite immune markers. By 45 min post-exercise, platelet counts had returned to baseline, neutrophil counts continued to rise (HIIRE > LIRE), and lymphocyte counts fell below baseline (HIIRE < LIRE). Those changes led to significant differences between HIIRE and LIRE 45-min post-exercise for all composite immune markers. Although interindividual variability was observed, individual response trajectories generally aligned with the overall group-level pattern, supporting the robustness of the observed responses within the crossover design.

This pattern aligns with what is generally observed during high-intensity exercise, where sympathetic activation and catecholamine release are thought to contribute to a rapid mobilisation of leukocytes and platelets (Foster et al. 1986; Benschop et al. 1996; de Teixeira et al. 2014; Joisten et al. 2019), whereas subsequent marrow release and slower clearance contribute to a more prolonged neutrophilia (Pedersen and Hoffman-Goetz 2000). Nonetheless, the differing kinetics between HIIRE and LIRE raise interesting questions about activation pathways and underlying mechanisms.

The substantial increase in HR observed in both *Conditions* (but superior in HIIRE) reflects sympathetic activation. Activation of the sympathetic nervous system leads to a rise in the release of epinephrine and norepinephrine, which bind to β2-adrenergic receptors on immune and endothelial cells, inducing a widespread cellular mobilisation (Mueller 2022). Specifically, lymphocytes are demarginated from the endothelial surface of blood vessels and released from secondary lymphoid organs (Pedersen and Hoffman-Goetz 2000). This preferentially mobilises β2-receptor-rich subsets, notably cytotoxic T lymphocytes (CD8⁺) and natural killer (NK) cells (Rumpf et al. 2021). Similarly, adrenergic stimulation demarginated neutrophils from the pulmonary and vascular endothelium into circulation (Dahdah et al. 2022). Platelets also express adrenergic receptors and can therefore, as well, be demarginated through this adrenergic surge (Barale et al. 2023). In addition, the spleen, which serves as a major platelet reservoir, is activated by norepinephrine binding to α1-adrenergic receptors on splenic smooth muscles, inducing splenic contraction, leading to the rapid release of platelets into circulation (Heber and Volf 2015). This results in a transient thrombocytosis immediately after exercise, reflecting sympathetic mobilisation of platelet reserves (Heber and Volf 2015).

Research has already widely shown that the production of catecholamines during exercise is intensity-dependent (Nieman 1997; Hackney 2006). The *Condition* differences immediately post-exercise for platelets (*d* = 1.356, *p* < .001) and for lymphocytes (*d* = 0.960, *p* < .001), in favour of HIIRE, and the moderate correlations between HR_mean_ and lymphocyte and platelet delta values from pre- to immediately post-exercise (Supplementary Material 1, Fig. S1) support this intensity-dependent effect. Nonetheless, the absence of detectable differences in the composite indexes suggests that their numerator and denominator are modulated to a comparable extent. This reinforces the idea that a common mobilisation pathway acts in parallel on multiple components of these composite indices, supporting catecholamine-mediated demargination as a key driver of the immediate changes observed.

At 45 min post-exercise (T_2_), HIIRE induced markedly greater increases in PLR, NLR and SII than LIRE (*d's* > 0.878), indicating a delayed but stronger systemic immune activation. At the level of individual components, both *Conditions* showed declines in lymphocyte and platelet counts from immediately post-exercise to 45 min post-exercise. This can be attributed to the cessation of catecholamine stimulation immediately after exercise (Pedersen and Hoffman-Goetz 2000). In addition to the stop of this sympathetic drive, exercise induces a dose-dependent increase in cortisol, which itself decreases systemic lymphocyte count (Nieman 1997; Hackney 2006). This lymphopenia 45 min post-exercise likely represents redistribution of lymphocytes to peripheral tissues following their initial mobilisation (Nieman 1997; Hackney 2006). Supporting this argument, previous research has shown that intense exercise elevates oxidative stress and mobilises mesenchymal stem cells from bone marrow, which in turn promote matrix metalloproteases (MMP) activity (Stamenkovic 2003; Gieseg et al. 2018). MMP-2 and MMP-9, then, can remodel the extracellular matrix by degrading basement-membrane components (e.g., the laminin-related extracellular matrix component, Netrin-4) and releasing matrix-bound chemokines, thereby facilitating cell movement through structural barriers (Stamenkovic 2003) and partly explain the transient post-exercise diapedesis of lymphocyte subsets. Because blood sampling ended 45 min post-exercise, the present study cannot determine the timing of peak NLR, PLR, or SII responses nor the duration required for their return to baseline. However, previous exercise-immunology studies suggest that lymphocyte counts generally recover within three to six hours post-exercise (Nieman 1997; Nieman and Wentz 2019), whereas neutrophilia may persist longer depending on exercise intensity and duration. Therefore, the elevated composite indices observed 45 min post-exercise (T_2_) likely represent an ongoing recovery process rather than the maximal or terminal phase of the response (Walzik et al. 2021). In the context of resistance exercise, it is generally less pronounced and less durable than following endurance exercise, likely due to a lower sympathetic activation and catecholaminergic stimulus (Simonson and Jackson 2004; Schlagheck et al. 2020; Walzik et al. 2021). Based on this rationale and considering the greater cardiovascular and metabolic responses observed during HIIRE, it can be hypothesised that the inflammatory markers measured following LIRE would return to baseline sooner than those observed following HIIRE. Further studies are warranted to confirm this hypothesis.

The 45-min post-exercise response was also intensity-dependent as evidenced by moderate inverse correlations between on the one hand HR_mean_ and cLac_max_ during exercise, and on the other hand platelet and lymphocyte delta values from immediately post-exercise to 45 min post-exercise (Supplementary Material 1, Fig. S2). The cortisol mediated dose response also explains the sustained increase in neutrophils, observed 45 min post-exercise, as cortisol promotes the recruitment of neutrophils from the bone marrow (Pyne 1994). Besides, the mobilisation of neutrophils from the bone marrow may also be triggered by the delayed action of lactate produced by muscles via G-protein-coupled receptor 81 signalling on endothelial cells, which increases vascular permeability and the release of neutrophil-mobilising factors (Khatib-Massalha et al. 2020). Indeed, the muscle can also influence the immune response and acts as an active endocrine organ, releasing myokines such as IL-6, IL-8, IL-1ra or TNF-α, which shape the immune profile 45 min post-exercise; for example, through neutrophil recruitment and lymphocyte diapedesis (Pedersen and Febbraio 2012; Ringleb et al. 2024). The significant difference in cLac between HIIRE and LIRE confirms greater glycolytic and muscular activation during HIIRE compared to LIRE (Campbell and Turner 2018; Estruel-Amades et al. 2019), suggesting a likely difference in myokine release and a stronger stimulation of sympathetic outflow.

Another often underestimated factor that may explain the kinetic differences between the immediate (T_1_) and 45-min post-exercise (T_2_) phases is the lymphatic system. During exercise, sympathetic activation and the mechanical muscle pump accelerate venous and lymphatic return, transiently flushing lymphocytes into the circulation through shear-stress-induced endothelial detachment and diapedesis (Nieman 1997; Pedersen and Febbraio 2012). When contractions cease, lymph flow and muscular pumping decline, while rising cortisol and chemokine gradients promote lymphocyte re-sequestration into lymphoid tissues—a process of diapedesis and homing that contributes to the post-exercise lymphopenia observed in early recovery (Peake et al. 2017; Campbell and Turner 2018; Estruel-Amades et al. 2019). The lymphatic network thus functions as a transient staging compartment for adaptive immune cells, modulating their redistribution between blood and lymphoid compartments (Kataru et al. 2019; Arasa et al. 2021). In HIIRE, this effect is intensified by the substantially higher metabolic and sympathetic load, which sustains cytokine signalling (IL-6, TNF-α), reactive oxygen species formation, and platelet activation beyond the cessation of exercise (Fatouros et al. 2010; Ringleb et al. 2024). The resulting combination of persistent neutrophilia and thrombocytosis, along with declining lymphocyte counts, amplifies PLR, NLR, and SII during early recovery. Consequently, *Condition* differences in NLR, PLR and SII become apparent only during early recovery at 45 min post-exercise (T_2_), when sustained sympathetic activation, muscle-derived cytokine release, and reactive oxygen species induced significant changes in neutrophils, lymphocytes and platelets.

From an exercise prescription perspective, the present findings suggest that intensity is a key modulator of the acute immune response to resistance exercise. Repeated acute changes could contribute to the long-term anti-inflammatory adaptations associated with regular exercise and higher-intensity resistance exercise may provide a stronger stimulus for such adaptations. As the present study assessed only acute responses, future longitudinal studies are needed to determine whether the greater immune activation observed following HIIRE translates into clinically meaningful benefits. Besides, it remains to be clarified whether the greater physiological stimulus induced by HIIRE is compatible with long-term adherence to exercise programmes, particularly in clinical populations, or whether a combination of higher- and lower-intensity resistance exercise modalities may help to better program recovery and optimise both immune adaptations and participant adherence.

A major strength of this randomised crossover trial lies in its rigorous methodological control and the use of a homogeneous, RE-trained male cohort, minimising interindividual variability in immune and metabolic responsiveness. Both exercise *Conditions* were matched for total relative volume and session duration, with only an average time difference of 3 min and 24 s between the two *Conditions* due to uncertainty in reaching MMF in the HIIRE, allowing the observed effects to be attributed primarily to exercise intensity. Standardisation of circadian timing, nutritional state, and abstinence from confounding behaviours (e.g., caffeine, alcohol, or prior exercise) ensured high internal validity. Moreover, the trial employed baseline-adjusted repeated-measures ANCOVAs and manipulation controls, including cLac and continuous HR monitoring, to verify that the manipulation of metabolic and cardiovascular load was successful. This integrated design provides one of the first controlled comparisons of intensity-dependent immune activation in RE, highlighting the role of metabolic strain and lymphatic redistribution in shaping short-term post-exercise immune responses.

Nonetheless, several limitations should be acknowledged. First, only routine immune markers (i.e., platelets, lymphocytes, neutrophils) and composite indices (i.e., NLR, PLR, SII) were analysed, which, while clinically relevant, do not capture the full spectrum of immune cell subsets, cytokines, or lymphatic mediators involved in post-exercise immune modulation. Including markers such as myokines (e.g., IL-6, TNF-α, IL-10), cortisol, catecholamines (e.g., epinephrine and norepinephrine), and lymphatic markers (e.g., soluble VEGF receptor-3 or Lymphatic vessel endothelial hyaluronan receptor-1) would allow a more detailed characterisation of the molecular mechanisms underlying 45-min post-exercise immune activation. Moreover, detailed baseline immunological profiling, including total IgE assessment or specific screening for subclinical allergic conditions, was not performed. Nonetheless, one can note that the manuscript reporting the primary outcomes of this trial (Javelle et al., *in prep*), will provide data on kynurenine metabolites and a deeper immune profiling. Second, as cLac was only a manipulation check marker, it was measured only during the exercise bout and up to five minutes post-exercise, but not 45 min post-exercise, preventing assessment of metabolic dynamics during the recovery window when immune differences emerged. Third, as the cohort consisted solely of young, RE-trained men, the findings may not generalise to untrained individuals, women, or older adults. Fourth, although the study was well-powered, the minimum detectable effect for 3 × 2 ANOVAs (with *β* = .80, *α* = .05, *ρ* = .50—sensitivity analysis G*Power 3.1.9.2) was small to moderate (*η*^*2*^_*p*_ = .034); therefore, it must be acknowledged that smaller effects may have gone undetected. Fifth, while standardisation was extensive, many of those variables relied on participants’ willingness to report, and one cannot exclude residual confounding from unreported physical activity or nutritional factors in the preceding 24 h. Finally, the present findings should be interpreted within the temporal limits of the sampling protocol. As measurements ended 45 min post-exercise, the observed differences reflect the early recovery phase only and do not permit conclusions regarding the duration or longer-term consequences of these immune responses. Future studies should therefore incorporate later measurement timepoints (e.g., 3, 6, 24 h post-exercise) with extended metabolic and cytokine profiling, post-exercise cLac sampling, and assessments of lymphatic function to validate the proposed model linking metabolic stress, lymphatic buffering, and delayed immune activation following high-intensity RE.

## Conclusion

This randomised crossover trial demonstrates that exercise intensity is a key determinant of the acute immune response to RE. While both HIIRE and LIRE elicited transient increases in circulating immune markers immediately post-exercise, only HIIRE induced a pronounced delayed elevation in NLR and SII 45 min post-exercise. These findings indicate that high-intensity RE provokes sustained immune activation extending into early recovery, likely driven by greater metabolic and sympathetic stress. Mechanistically, the results support a model in which intense muscular contractions enhance neutrophil and platelet mobilisation while subsequent lymphocyte diapedesis and lymphatic sequestration amplify ratio-based inflammation indices during recovery. The lymphatic system may act as a transient staging compartment, helping the redistribution of immune cells after high-intensity RE. Together, these data provide new evidence that not only the volume but also the intensity and recovery kinetics of RE critically shape the magnitude and timing of the cellular inflammatory response. Future trials integrating endocrine, cytokine, and lymphatic measures are warranted to elucidate the interplay between metabolic stress, immune cell trafficking, and systemic inflammation across diverse patient populations and training contexts in both acute and long-term settings.

## Supplementary Information

Below is the link to the electronic supplementary material.

Supplementary Material 1

## Data Availability

The protocol of this trial with directional hypotheses together with raw data was uploaded to Open Science Framework (https://doi.org/10.17605/OSF.IO/E75N9).

## Abbreviations

AUDIT: Alcohol Use Disorders Identification Test
ANCOVA: Analysis of covariance
BIA: Bioelectrical Impedance Analysis
bpm: Beats per minute
cLac: Capillary lactate
CI: Confidence Interval
CONSORT: Consolidated Standards of Reporting Trials
EE: Endurance exercise
FFMI: Fat-free mass index
HIIRE: High-intensity interval resistance exercise
HR: Heart rate
IL: Interleukin
LIRE: Low-intensity resistance exercise
MMF: Momentary muscular failure
MMP: Matrix metalloproteases
NLR: Neutrophil-to-lymphocyte ratio
PLR: Platelet-to-lymphocyte ratio
RE: Resistance exercise
RM: Repetition maximum
SII: Systemic immune-inflammation index
WBC: White blood cell
