# Supplementary material for: Intensity matters: high-intensity interval resistance exercise induces greater immune activation than low-intensity resistance exercise 45 min post-workout
Source: Eur J Appl Physiol. 2026 Jul 16;126(10):5579–94. doi: 10.1007/s00421-026-06337-z (PMC13633966; doi:10.1007/s00421-026-06337-z)
Supplement: Supplementary file 1 [file 421_2026_6337_MOESM1_ESM.docx]

**Supplementary Material**

**Intensity matters: High-Intensity Interval Resistance Exercise Induces Greater Delayed Immune Activation Than Low-Intensity Resistance Exercise**

Fabian Fabritius^a^, Matthias Rißmayer^a,b^, Miriam Ringleb^a,c,d^ Niklas Joisten^e^, Wilhelm Bloch^f^, Finn Dreisigacker^a^, Florian Javelle^a*^

**Supplementary Note 1:** *Standardised range of motion criteria*

Range of motion was standardized across participants, exercises, and conditions. Specifically, a controlled 90° knee angle was required in the back squat, the barbell was required to touch the chest in the bench press, the barbell had to be lowered below knee level in the Romanian deadlift, and the barbell had to be lowered below chin level in the overhead press. All exercises were performed through the full positive (concentric) range of motion, and repetitions were executed continuously without any rest between reps.

**Table S1:** Computation of 6RM performance score

| Performance level | 6RM  Squats  [% body mass] | 6RM  Bench Press  [%body mass] | 6RM  Deadlift  [%body mass] | 6RM  Overhead Press  [%body mass] |
| --- | --- | --- | --- | --- |
| Intermediate  (1 point) | ≤ 102 | ≤ 85 | ≤ 127.5 | ≤ 50 |
| Advanced  (2 points) | ≤ 127.5 | ≤ 102 | ≤ 153 | ≤ 70 |
| Highly Advanced  (3 points) | > 127,5 | > 102 | > 153 | > 70 |

*Note.*

Points to be awarded for calculating the individual total 6RM performance score are presented. Threshold values for Squats, Bench Press and Deadlifts were set based on Santos Junior and colleagues (Santos Junior et al., 2021), while due to lack of reference data for the Overhead Press threshold values were set based on pilot runs. Based on the 6RM performance level (in % of individual body mass) 1 (intermediate), 2 (advanced) to maximal 3 points (highly advanced) were awarded for each of the 4 exercises. Minimum total 6RM performance score is 4 out of 12 possible points and 12 out of 12 possible points the maximum reachable 6RM performance score. A total 6RM performance score from 4-6 was set as Intermediate, 7-9 as Advanced and 10-12 as Highly Advanced. The individual total 6RM performance score was used as a stratification factor for randomisation.

**Table S2:** 6RM performance characteristics

|  | | Overall | HIIRE+LIRE | LIRE+HIIRE | Independent Samples  T-Test |
| --- | --- | --- | --- | --- | --- |
|  |  | *n* = 24 | *n* = 11 | *n* = 13 | *p* |
|  | 6RM Squats  [% body mass] | 125.7 ±21.4 | 117.2 ±12.8 | 132.9 ±24.8 | .071 |
|  | 6RM Bench Press  [% body mass] | 99.0 ±13.6 | 102.0 ±13.6 | 96.5 ±13.6 | .338 |
|  | 6RM Deadlift  [% body mass] | 148.1 ±22.3 | 147.5 ±16.0 | 148.6 ±27.2 | .906 |
|  | 6RM Overhead Press  [% body mass] | 61.9 ±8.6 | 62.6 ±8.2 | 61.2 ±9.1 | .709 |
|  | Total 6RM Score  (min. 4 – max. 12) | 8.8 ±1.6 | 8.6 ±1.4 | 8.9 ±1.7 | .664 |

*Note.*

Means ± SDs for 6RM performance characteristics are presented. Bolded and asterisked areas mark significant differences between group allocation *(i.e., HIIRE first vs LIRE first)* at *p* < .05 (*), *p* < .01 (**) and *p* < .001 (***). 6RM: 6-repetition maximum; Intervention sequences: HIIRE+LIRE: HIIRE followed my LIRE; LIRE+HIIRE: LIRE followed by HIIRE.

**Table S3:** Randomisation order, stratification factors and group allocation

| Participant-ID | Randomisation order | FFMI [kg · m²] | Total 6RM score | Group |
| --- | --- | --- | --- | --- |
| MF_01 | 1 | 18.78 | 9 | HIIRE first |
| MF_02 | 2 | 20.63 | 6 | HIIRE first |
| MF_03 | 3 | 21.57 | 10 | LIRE first |
| MF_04 | 4 | 22.80 | 6 | LIRE first |
| MF_05 | 9 | 23.31 | 11 | HIIRE first |
| MF_06 | 7 | 22.45 | 9 | LIRE first |
| MF_07 | 5 | 23.05 | 10 | HIIRE first |
| MF_08 | 14 | 19.97 | 8 | HIIRE first |
| MF_09 | 15 | 23.22 | 9 | HIIRE first |
| MF_10 | 6 | 21.93 | 11 | HIIRE first |
| MF_11 | 8 | 20.67 | 9 | LIRE first |
| MF_12 | 11 | 21.49 | 8 | HIIRE first |
| MF_13 | 10 | 18.79 | 9 | LIRE first |
| MF_14 | 12 | 19.02 | 12 | LIRE first |
| MF_15 | 13 | 23.98 | 11 | LIRE first |
| MF_16 | 17 | 19.40 | 9 | HIIRE first |
| MF_17 | 20 | 19.16 | 7 | HIIRE first |
| MF_18 | 24 | 18.93 | 8 | LIRE first |
| MF_19 | 16 | 18.54 | 8 | LIRE first |
| MF_20 | 23 | 19.17 | 8 | HIIRE first |
| MF_21 | 18 | 19.52 | 9 | LIRE first |
| MF_22 | 25 | 20.74 | 9 | LIRE first |
| MF_23 | 21 | 17.82 | 10 | LIRE first |
| MF_24 | 22 | 22.53 | 6 | LIRE first |
| MF_25 | 19 | 21.49 | 10 | HIIRE first |

*Note.*

Values for randomisation order, stratification factors (FFMI, total 6RM score) and group allocation *(i.e., HIIRE first vs LIRE first)* are presented. Participant ID was not included for any further tests / means. Red marked participant was excluded due to an intervention-independent injury. For randomisation stratification factors were divided into different levels. FFMI: “Low to normal muscle mass”: < 20; “muscular above average”: ≥ 20; “very muscular”: ≥ 22. Total 6RM performance score: Intermediate: 4-6; Advanced: 7-9; Highly Advanced: 10-12.

**Table S4:** *Raw values for neutrophils, lymphocytes, platelets and cellular inflammatory markers*

|  | HIIRE | | | LIRE | | |
| --- | --- | --- | --- | --- | --- | --- |
|  | ***n* = 24** | | | ***n* = 24** | | |
|  | **T_0_** | **T_1_** | **T_2_** | **T_0_** | **T_1_** | **T_2_** |
| NEU [10^3^/µl] | 3.00  ±0.92 | 4.32  ±1.79 | 5.58  ±2.27 | 2.95  ±0.93 | 3.85  ±1.96 | 4.05  ±1.74LY |
| LYM [10^3^/µl] | 2.17  ±0.40 | 2.42  ±0.72 | 1.48  ±0.30 | 2.15  ±0.58 | 2.06  ±0.56 | 1.71  ±0.41 |
| PLT [10^3^/µl] | 224.38  ±45.92 | 253.87  ±47.01 | 232.15±  44.97 | 222.27  ±39.38 | 236.66  ±42.16 | 228.42  ±43.66 |
| NLR | 1.44  ±0.53 | 1.94  ±0.95 | 3.99  ±2.12 | 1.47  ±0.7 | 1.89  ±0.93 | 2.56  ±1.44 |
| PLR | 108.16  ±36.11 | 115.00  ±43.36 | 162.20  ±41.83 | 110.35  ±35.91 | 124.35  ±45.94 | 141.76  ±38.86 |
| SII | 327.79  ±151.04 | 504.41  ±290.43 | 944.14  ±552.62 | 316.44  ±160.21 | 461.13  ±277.5 | 603.19  ±399.00 |

*Note.*

Means ± SDs of white blood cells (WBCs), neutrophils (NEU), lymphocytes (LYM), platelets (PLT) and cellular inflammatory markers (NLR, PLR, SII) are presented before data transformation. One missing data point was imputed (see section 2.9 of the manuscript). Data sets were winsorised ± 3 SDs. NLR, PLR and SII were calculated based on NEU, LYM and PLT counts determined via haematology analysis and the resulting complete blood count (see section 2.7.1 of the manuscript). T_0_ : baseline ; T_1_ : immediately post-exercise ; T_2_ : 45 minutes post-exercise. NLR = NEU/LYM ; PLR = PLT/LYM ; SII = (PLT × NLR).

**Table S5:** *Participant-specific intervention characteristics*

|  | Independent Samples T-Test by *Group* | |
| --- | --- | --- |
|  | ***p*** | ***Cohen’s d*** |
| Relative Volume (Repetitions · % 1RM) | .098 | 0.489 |
| Intervention Duration | .129 | 0.458 |
| HR_mean_ [bpm] | .070 | 0.549 |
| \| .031* \| 0.017 | **Paired Samples T-Test by *Condition*** | |
|  | ***p*** | ***Cohen’s d*** |
| Relative Volume (Repetitions · % 1RM) | .691 | 0.082 |
| Intervention Duration | **< .001***** | **1.246** |
| HR_mean_ [bpm] | **< .001***** | **0.988** |

*Note.*

Independent Samples and Paired Samples T-Test outcomes for intervention characteristics: relative volume (Repetitions · % 1RM), intervention duration and average heart rate (HR_mean_) are presented. Bolded and asterisked areas mark significant differences between *Groups* *(i.e., HIIRE first vs LIRE first)* or *Conditions* *(i.e., HIIRE vs LIRE)* at p < .05 (*), p < .01 (**) and p < .001 (***).

**Table S6:** *Baseline-adjusted means of capillary lactate according to Time and Condition*

| HIIRE | L0 | L1 | L2 | L3 | L4 | L5 | L6 |
| --- | --- | --- | --- | --- | --- | --- | --- |
| cLac [mmol/L] | 1.029 | 1.684  ± 0.102 | 7.155  ± 0.511 | 8.341  ± 0.429 | 8.251  ± 0.451 | 7.990  ± 0.360 | 6.646  ± 0.400 |
| LIRE | **L0** | **L1** | **L2** | **L3** | **L4** | **L5** | **L6** |
| cLac [mmol/L] | 1.029 | 1.601  ± 0.102 | 5.825  ± 0.511 | 6.764  ± 0.429 | 5.925  ± 0.451 | 6.695  ± 0.360 | 5.308  ± 0.400 |

*Note.*

Adjusted means ± SEMs for capillary lactate (cLac) are presented and were determined via baseline-adjusted repeated measures ANCOVAs. Depicted values are shown in baseline-adjusted line charts (see Figure 3). Time points for cLac: L0: pre warm-up; L1: post warm-up; L2: post Squats; L3: post Bench Press; L4: post Deadlift; L5: post Overhead Press; L6: 5 minutes post-exercise.

**Table S7:** A-posteriori power analyses

| Variable | *η^2^_p_* [95% CI]  for Time x Condition | Power  [95% CI] |
| --- | --- | --- |
| NEU | .145 [0.053 to 1.000] | 98.40% [97.41to 99.08%] |
| LYM | .155 [0.060 to 1.000] | 98.70% [97.79 to 99.31%] |
| PLT | .121 [0.037 to 1.000] | 94.50% [92.90 to 95.83%] |
| NLR | .226 [0.117 to 1.000] | 99.90% [99.44 to 100%] |
| PLR | .107 [0.028 to 1.000] | 90.40% [88.40 to 92.15%] |
| SII | .174 [0.075 to 1.000] | 99.30% [98.60 to 99.7%] |

*Note.*

*η^2^_p_* for *TimexCondition* and a-posteriori power for neutrophils (NEU), lymphocytes (LYM), platelets (PLT) and cellular inflammatory markers (Neutrophil-to-Lymphocyte Ratio [NLR], Platelet-to-Lymphocyte Ratio [PLR], Systemic Immune-Inflammation Index [SII]) are presented. NLR, PLR and SII were calculated based on NEU, LYM and PLT counts determined via haematology analysis and the resulting complete blood count (see section 2.7.1 of the manuscript). A-posteriori power-analyses were performed using the *simr* package on R for *TimexCondition* with 1000 simulations.

**Fig. S1:** *During exercise: Pearson’s r correlations of maximum capillary lactate and average heart rate with changes in cell counts and cellular inflammatory markers*

Note. Pearson’s r values are presented (range from -1.00 to 1.00). Delta (Δ) values for percental changes from T_0_ to T_1_ in cell counts and cellular inflammatory markers were correlated with cLac_max_ and HR_mean_. Green areas mark a significant positive correlation. (r is positive). Red areas indicate a significant negative correlation (r is negative), where an increase in one variable corresponds to a decrease in the other. White areas (r = 0) indicate no correlation, i.e. there is no linear relationship between the variables. cLac_max_: maximum capillary lactate; HR_mean_: average heart rate.

**Fig. S2:** *Post-exercise: Pearson’s r correlations of maximum capillary lactate and average heart rate with changes in cell counts and cellular inflammatory markers*

Note. Pearson’s r values are presented (range from -1.00 to 1.00). Delta (Δ) values for percental changes from T_1_ to T_2_ in cell counts and cellular inflammatory markers were correlated with cLac_max_ and HR_mean_. Green areas mark a significant positive correlation. (r is positive). Red areas indicate a significant negative correlation (r is negative), where an increase in one variable corresponds to a decrease in the other. White areas (r = 0) indicate no correlation, i.e. there is no linear relationship between the variables. cLac_max_: maximum capillary lactate; HR_mean_: average heart rate.
